# Characterization and Vector Competence Studies of Chikungunya Virus Lacking Repetitive Motifs in the 3′ Untranslated Region of the Genome

**DOI:** 10.3390/v13030403

**Published:** 2021-03-04

**Authors:** Yauhen Karliuk, Anja vom Hemdt, Janett Wieseler, Martin Pfeffer, Beate M. Kümmerer

**Affiliations:** 1 Institute of Animal Hygiene and Veterinary Public Health, Faculty of Veterinary Medicine, University of Leipzig, 04103 Leipzig, Germany; yauhen.karliuk@vetmed.uni-leipzig.de (Y.K.); pfeffer@vetmed.uni-leipzig.de (M.P.); 2 Institute of Virology, Medical Faculty, University of Bonn, 53127 Bonn, Germany; Anja.vom_Hemdt@ukbonn.de (A.v.H.); Janett.Wieseler@ukbonn.de (J.W.)

**Keywords:** chikungunya virus, vector competence, 3′ untranslated region, direct repeats, *Aedes vexans*, *Culex pipiens*

## Abstract

Using reverse genetics, we analyzed a chikungunya virus (CHIKV) isolate of the Indian Ocean lineage lacking direct repeat (DR) elements in the 3′ untranslated region, namely DR1a and DR2a. While this deletion mutant CHIKV-∆DR exhibited growth characteristics comparable to the wild-type virus in Baby Hamster Kidney cells, replication of the mutant was reduced in *Aedes albopictus* C6/36 and *Ae. aegypti* Aag2 cells. Using oral and intrathoracic infection of mosquitoes, viral infectivity, dissemination, and transmission of CHIKV-∆DR could be shown for the well-known CHIKV vectors *Ae. aegypti* and *Ae. albopictus*. Oral infection of *Ae. vexans* and *Culex pipiens* mosquitoes with mutant or wild-type CHIKV showed very limited infectivity. Dissemination, transmission, and transmission efficiencies as determined via viral RNA in the saliva were slightly higher in *Ae. vexans* for the wild-type virus than for CHIKV-∆DR. However, both *Ae. vexans* and *Cx. pipiens* allowed efficient viral replication after intrathoracic injection confirming that the midgut barrier is an important determinant for the compromised infectivity after oral infection. Transmission efficiencies were neither significantly different between *Ae. vexans* and *Cx. pipiens* nor between wild-type and CHIKV-∆DR. With a combined transmission efficiency of 6%, both *Ae. vexans* and *Cx. pipiens* might serve as potential vectors in temperate regions.

## 1. Introduction

Chikungunya virus (CHIKV) is a re-emerging arbovirus that is transmitted to humans through anthropophilic mosquitoes of the *Aedes* genus [1]. The term chikungunya means ‘to walk bent over’ in some east African languages and is attributed to the joint pains which occur during infection [2]. Besides myalgia and arthralgia, high fever, headache, and rash are common symptoms of chikungunya disease [2]. 

CHIKV was first isolated in 1953 in the Newala district of Tanzania [3]. The virus came back into focus due to an urban epidemic in 1999–2000 in Kinshasa, Democratic Republic of the Congo, with about 50,000 infected people [4]. In 2004 an outbreak was reported on the island Lamu, Kenya, which affected almost 75% of the island population [5] before the virus spread in 2005 to further islands in the Indian Ocean including the Grande Comoro Island, La Réunion, Mayotte, Mauritius, and the Seychelles [6,7,8]. The virus subsequently reached India where over 1.3 million infected people were reported during 2005–2006 [9,10]. The epidemic on La Réunion which affected almost 250,000 people is particularly noteworthy as it is the first documented report of a CHIKV outbreak in which *Aedes albopictus* was the main vector in Africa. A mutation in the CHIKV E1 protein (E1-A226V) was found to yield a higher fitness and better transmission of CHIKV in *Ae. albopictus* mosquitoes [11,12]. Subsequently, CHIKV outbreaks involving *Ae. albopictus* were also reported in temperate regions. In 2007, first local outbreaks with the E1-A226V variant were reported in the province of Ravenna, Italy [13]. The adaptive mutation was also present during an autochthonic outbreak in south-east France in 2017 [14]. In late 2013 CHIKV emerged in the Caribbean where transmission occurred by *Ae. aegypti* resulting in over 2.5 million cases until the end of 2017 in the Americas [15,16]. Phylogenetic analyses revealed that the virus causing the Caribbean epidemics belongs to the Asian genotype [17].

CHIKV is an enveloped, positive-strand RNA virus of the genus Alphavirus within the family *Togaviridae*. The genome is capped at the 5′ end and polyadenylated at the 3′ end. It has a length of about 12 kilobases and encodes two open reading frames (ORF) [18]. The first ORF, encompassing the 5′ two-thirds of the genome, encodes the nonstructural proteins nsP1-nsP4. The second ORF, present in the last third of the genome, encodes the structural proteins (capsid, E1, and E2) and two small cleavage products (E3 and 6k). The ORFs are flanked by two untranslated regions (UTRs). The alphavirus 5′ UTR contains cis-acting elements involved in regulating minus and plus strand RNA synthesis [19]. The 3′ UTRs are characterized by several repeated sequence elements and a conserved sequence element (CSE) directly upstream of the poly(A) tail [20,21,22]. 

CHIKV can be classified into three main genotypes: East, Central, and South African (ECSA), West African (WA), and Asian genotype [23]. Within the ECSA genotype, the Indian Ocean (IO) lineage emerged [23], whereas CHIKV isolates from the 2013 Caribbean outbreak from a novel American lineage within the Asian genotype [24]. For CHIKV, the number of direct repeats (DRs 1, 2, and 3) and their arrangement differs between CHIKV lineages [22,25]. Compared to the WA and ECSA genotypes, members of the Asian genotype have a longer 3′ UTR, encompassing more DR elements. The emergence of the different UTRs has been described to be an evolutionary process shaped by fitness trade-offs and population bottlenecks [22,26]. Experimental deletion of some DR elements within the CHIKV 3′ UTR of a Caribbean strain resulted in reduced replication in mosquito cells and delayed replication in *Ae. aegypti* and *Ae. albopictus* mosquitoes leading to a longer extrinsic incubation period [27]. This extrinsic incubation time describes the viral incubation period between the time when a mosquito takes a viremic bloodmeal and the time when the mosquito becomes infectious; it is positively correlated with temperature, but mainly depends on how good and fast the virus is able to cross tissue barriers within the mosquito. Tissue barriers at the midgut and salivary gland levels also play an important role with regard to vector competence in general [28]. While the midgut infection barrier (MIB) regulates whether a virus is able to enter and establish an infection in the midgut, the midgut escape barrier (MEB) affects the ability to escape from the midgut to allow dissemination in the mosquito secondary tissues [28]. Similarly, salivary gland infection and escape barriers (SGIB and SGEB) exist that must be overcome in order for the virus to be secreted in saliva and transmitted to the host. The CHIKV E1-A226V mutation mentioned before allowed the virus to cross more easily the MIB in *Ae. albopictus* [12,29]. The latter mosquito species is able to adapt very well to different climatic conditions. This ability, climate change, and globalization enabled an enormous spread of *Ae. albopictus* also in Europe [30,31,32,33,34]. This carries the risk that CHIKV will spread further, especially considering that global warming is playing an increasingly important role. Already in 2016, a successful overwintering of *Ae. albopictus* in Germany was reported [35].

Altogether, these findings also raise the question as to what extent other mosquitoes in temperate regions are able to transmit CHIKV. We therefore aimed to investigate on the one hand whether mosquitoes from the same genus as the main vectors *Ae. aegypti* and *Ae. albopictus* as well as also being present in temperature regions are able to serve as vector. One such mosquito species is *Aedes vexans*, which is distributed almost worldwide and therefore also present in Europe [34]. On the other hand, we were interested to analyze whether a further distantly related mosquito genus, namely *Culex*, is competent to transmit CHIKV. Since the 3′ UTR has been described to impact mosquito transmission [28], the respective studies were performed for a CHIKV with deletion of DR elements in the 3′ UTR as well as CHIKV wild-type virus. 

## 2. Materials and Methods

### 2.1. Mosquitoes

Four laboratory reared mosquito species were used in the experiments (Table 1). 

### 2.2. Rearing Mosquitoes

The mosquitoes were kept in 50 × 50 × 45 cm (length/width/height) cages at room temperature of 25 ± 1 °C and 65 ± 10% relative humidity. The following light-regime was used: 0.5 h twilight—16 h daylight—0.5 h twilight—7 h darkness. An 8% fructose solution was used as a maintaining food and liquid source for the mosquitoes. Feeding was carried out using cotton pads moistened with fructose solution, which were placed on the mosquito cages. For blood feeding we used expired erythrocyte concentrate from the transfusion medicine of the University Hospital Leipzig. For feeding the mosquitoes a parafilm-covered polyoxymethylene (POM) plate [36] was filled with 4 mL of erythrocyte concentrate-fetal bovine serum (FBS, Sigma-Aldrich, St. Louis, MO, USA) solution (1:1). The loaded POM plate was placed on the mosquito cage and kept at 37 °C using a size-fitting hot plate.

### 2.3. Cell Culture

Baby Hamster Kidney cells (BHK-21/J, kindly provided by Charles M. Rice, Rockefeller University, New York, NY, USA) were grown in MEM (Gibco, Thermo Fisher Scientific, Waltham, MA, USA) containing 7.5% FBS (Sigma, St. Louis, MO, USA), 1% L- glutamine and 1% non-essential amino acids (NEAA) (Gibco). Vero B4 cells were cultured in DMEM (Gibco) containing 1% L-glutamine and 10% FBS. Leibovitz’s L-15 Medium (Gibco) with 10% FBS (Sigma, St. Louis, MO, USA) was used to grow C6/36 cells (ATCC). Aag2 cells (CCL/FLI, Greifswald, Germany) were cultured in Leibovitz’s L-15 Medium (Gibco) supplemented with 10% FBS (Sigma, St. Louis, MO, USA), 10% tryptose phosphate broth solution (Sigma, St. Louis, MO, USA) and 1% NEAA. BHK and Vero cells were kept at 37 °C with 5% CO_2_. The insect cells were cultured at 28 °C without CO_2_. 

### 2.4. Construction of CHIKV cDNA Clones 

The CHIKV-WT used in this study was derived from a previously described Mauritius infectious cDNA clone, rCHIKV [37], in which a mutation for the E1-A226V exchange was introduced. To this end, two PCR fragments were amplified from the rCHIKV plasmid using primers Bo946 (5′-GAAGTGCTGCGGTACAGCAGA-3′) and Bo1082 (5′-TACCGTACCCACAGCCGGTCTCT-3′) or Bo1081 (5′-AGAGACCGGCTGTGGGTACGGTACA-3′) and Bo1083 (5′-CAGTCCAGTTACGCTGGAGTCTGAG-3′), respectively. The obtained fragments were fused via PCR amplification using the outer primers Bo946 and Bo1083. The resulting fragment was cut with *SgrA*I and *Not*I and inserted into the infectious rCHIKV clone cut with the same restriction enzymes. CHIKV-∆DR corresponds to a virus established by first amplifying two PCR fragments using primers Bo946 and Bo1474 (5′-TGTCTCTTAGGGGACACGTATACCTTCATACTTAATTGTCAAGTTAGTGCCT-3′) or Bo1473 (5′-AGGTATACGTGTCCCCTAAGAGACA-3′) and Bo1083, respectively from rCHIKV-WT (E1-A226V), followed by fusion PCR using the outer primers and thereafter replacing the *SgrA*I-*Not*I fragment in the rCHIKV-WT plasmid against the amplified PCR fragment cut with the same enzymes. Changes introduced into the plasmids were verified via Sanger sequencing. Using primers Bo1392 (5′-GCCATACTCTCAGGCACCAT-3′) and Bo1393 (5′-ATTAAAAACAAAATAACATCTCCTACGTCCCTGTG-3′), the 3′ UTR regions of the recombinant viruses were amplified via RT-PCR and subsequently sequenced to confirm the genotypes of CHIKV-WT and CHIKV-∆DR in this region. The deletion encompasses nucleotides (nt) 11332–11461 (according to GenBank number FJ959103). 

### 2.5. In Vitro Transcription and Recovery of Recombinant Virus

Infectious cDNA clones were linearized with *Not*I, and in vitro transcription was performed using the T7 mMessage Machine Kit (Invitrogen^TM^, Thermo Fisher Scientific, Waltham, MA, USA) according to the manufacturer’s instruction. The integrity and amount of the in vitro transcribed RNA were analyzed by electrophoresis in ethidium bromide agarose gels. To recover recombinant viruses, the in vitro transcribed RNA was electroporated into BHK cells as described previously [38]. For mosquito infection experiments virus was passaged once on Vero B4 cells. 

### 2.6. Growth Kinetic Studies in Cell Culture

Cells seeded the day before at 8 × 10^4^ (BHK) or 2.5 × 10^5^ (C6/36, Aag2) cells per 24 well were infected in triplicate at a multiplicity of infection (MOI) of 0.1 with wild-type or mutant CHIKV for 1 h at 37 °C or 28 °C, respectively. After infection, cells were washed once with PBS and once with media without supplements before adding the corresponding cell culture media. Aliquots of the supernatants were harvested at 0 h, 8 h, 20 h, 30 h, and 44 h (BHK and C6/36 cells) or 0 h, 24 h, 48 h, 72 h, and 96 h (Aag2 cells) and used to determine viral titers via plaque assay on BHK cells. 

### 2.7. Plaque Assay

Titration of virus stocks used for mosquito infection was performed on Vero B4 cells. Viral titers for assessment of growth curves were determined on BHK cells. Cells seeded the day before at 3 × 10^5^ cells (BHK) or 5 × 10^5^ cells (Vero B4) per 6-well were infected with 200 µL of ten-fold dilutions of the virus solution and incubated for 1 h at 37 °C. Thereafter, cells were covered with 3 mL overlay obtained by mixing 1.2% agarose with 2× MEM, 4% FBS, 2% Pen/Strep at a ratio of 1:1. After incubation for two days at 37 °C, cells were fixed with 6% formaldehyde and stained with crystal violet solution to visualize plaques. 

### 2.8. Infection of Mosquitoes

Infection of mosquitoes with CHIKV was performed in a Biosafety Level 3 laboratory at the University of Bonn Medical Centre. Prior to oral infection 5–10 days old female mosquitoes were starved for 24 h. Feeding was performed in groups of up to 20 individuals. Virus-spiked blood meal for infection was prepared essentially as described [39]. Briefly, human expired erythrocyte concentrate was mixed with 8% fructose solution, FBS and virus stock in a ratio of 5:3:1:1 so that the final concentration of virus solution was 1 × 10^6^ PFU/mL. This viral titer was chosen as it is in the range of viremic titers found in CHIKV patients [40,41]. Depending on the type of mosquito, two different oral infection methods were used. Oral infection of *Ae. aegypti* mosquitoes was performed using a Hemotek membrane feeding device (Hemotek Ltd., Blackburn, UK). For *Ae. vexans*, *Ae. albopictus*, and *Culex pipiens* (*Cx. pipiens*) oral infection was done with cotton swabs each soaked with 200 μL of blood-virus solution. Feeding was allowed for two to three hours. To determine blood fed mosquitoes, the insects were anesthetized on ice and visually inspected for blood uptake. In total, 27 *Ae. aegypti*, 20 *Ae. albopictus*, 78 *Ae. vexans*, and 62 *Cx. pipiens* engorged and were included in the experiments. Intrathoracic injection was performed according to Rosen and Gubler [42]. Female mosquitoes were anesthetized by cooling on ice and intrathoracally injected with 200 plaque forming units (PFU) of virus in 27.6 nL 0.9% sodium chloride solution (Braun, Melsungen, Germany). Injection was performed with a fine glass capillary (Drummond Scientific Company, Broomall, PA, USA) using a Nanoject II device (Drummond Scientific Company). Infected mosquitoes were kept in climate incubators at 28 °C and 80% humidity with a 12 h light–12 h dark photocycle. 

### 2.9. Mosquito Processing

Infected mosquitoes were either sampled as a whole or dissected anesthetized on ice into legs, wings, and body using a Leica DMS 1000stereomicroscope (Leica, Wetzlar, Germany). After removal of legs and wings, the proboscides of the mosquitoes were inserted into a 10 µL pipette tip filled with 5 µL of FBS. Then 1 µL of eye drops with 1% pilocarpine hydrochloride (Pilomann 1%, Bausch-Lomb, Rochester, NY, USA) was applied to the thorax of the mosquito to force salivation [43]. Salivation was allowed for 20–30 min before the content of the pipet tip was mixed with 15 µL of MEM cell culture media and stored at −80 °C until further processing. Whole mosquitoes or mosquito body parts were stored at −80 °C as well. 

### 2.10. Nucleic Acid Extraction from Mosquitoes and Real-Time RT-PCR

To extract viral RNA from whole mosquitoes or mosquito body parts, 300 µL MEM media without FBS and 8–10 Precellys zirconium oxide beads of 1.4 mm diameter (Bertin Technologies, Montigny-le-Bretonneux, France) were added to the respective samples. The tubes were placed into a tissue lyser adaptor and stored at −20 °C for 4–8 min before the samples were shredded twice for 30 s with a frequency of 30 Hz using a TissueLyser (Qiagen, Hilden, Germany). To remove remaining body parts, the samples were centrifuged at 4 °C for 10 min at 2500 rpm. Then, 140 µL of the supernatant was added to 600 µL RAV1 buffer (NucleoSpin RNA virus kit, Macherey-Nagel, Macherey-Nagel, Düren, Germany), heated for 10 min at 70 °C and proceeded for RNA isolation according to the manufacturer’s instructions (Macherey-Nagel). For real-time RT-PCR analyses of saliva, 10 µL of the stored saliva solution (see above) was mixed with 130 µL MEM media and processed using the Macherey-Nagel kit as described before. 

Viral genome copy numbers from processed mosquito samples were quantified by real-time RT-PCR in a One Step RT-PCR reaction (SuperScript™ III One-Step RT-PCR System with Platinum™ Taq DNA Polymerase, Invitrogen^TM^) using primers Bo1171 (5′-TGACCCCGACTCAACCATCCT-3′) and Bo1172 (5′-GGCAGACGCAGTGGTACTTCCT-3′) and probe Bo1179 (5′-6-carboxyfluorescein [FAM]-TCCGACATC/ZEN/ATCCTCCT TGCTGGC-lowa Black^®^FQ-3′). RT-PCR involved reverse transcription at 50 °C for 30 min, initial denaturation at 94 °C for 2 min, and 45 cycles with 94 °C for 15 s, 58 °C for 30 s and 72 °C for 30 s. In vitro transcribed RNA from the target region served as standard for each separate run. A representative standard curve used for quantification is shown in Figure S1. Samples were run in a LightCycler^®^ 480 Instrument II (Roche, Basel, Switzerland). 

### 2.11. Vector Competence Indices

The infection rate after oral feeding is the number of CHIKV real-time RT-PCR positive carcasses in relation to the total number of mosquitoes examined. The dissemination rate refers to the number of real-time RT-PCR positive wings (or wings and legs) in relation to the numbers of positive carcasses. The transmission rate is the percentage of mosquitoes with positive carcasses and wings (or wings and legs) that were also positive in the saliva. The transmission efficiency corresponds to the number of mosquitoes that were tested positive in the saliva in relation to the total number of mosquitoes examined.

### 2.12. Statistics

Data were analyzed using GraphPad Prism 6 (GraphPad Software Inc., San Diego, CA, USA). If needed, values were normalized to d0 values prior to calculation. Differences of viral replication in *Ae. vexans* and *Cx. pipiens* after intrathoracic infection with either CHIKV-WT or CHIKV-∆DR were calculated using the Mann–Whitney test. Comparison of transmission efficiencies in these mosquito species after oral infection was calculated with Fisher’s exact test. For both tests a *p* < 0.05 was considered significant (* *p* < 0.05; ** *p* < 0.01; *** *p* < 0.001; **** *p* < 0.0001).

## 3. Results

### 3.1. Characterization of CHIKV Lacking DR Elements in the 3′ UTR in Cell Culture

**Figure 1:**
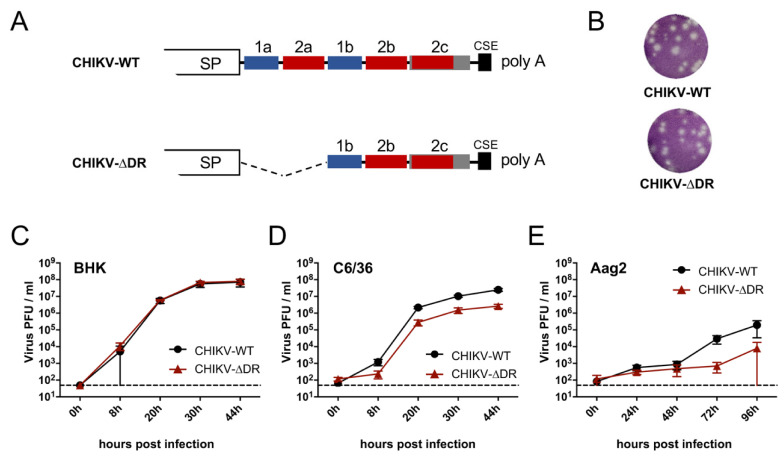
Growth kinetics of wild-type and ∆DR mutant CHIKV in different cells. (**A**) Schematic drawing of the 3′ UTRs of wild-type and mutant viruses. For CHIKV-∆DR, 130 nt encompassing the DR elements 1a and 2a were deleted. SP = structural protein coding region: CSE = 19 nt long conserved sequence element directly adjacent to the poly A tail. (**B**) Plaque morphology of CHIKV-WT and CHIKV-∆DR in Vero cells. Infected cells were overlaid with agarose. At two days post infection, cells were fixed and subjected to crystal violet staining. (**C**–**E**) Growth kinetics of CHIKV-WT and CHIKV-∆DR in vertebrate (BHK) and mosquito (C6/36, Aag2) cells. Cells were infected at an MOI of 0.1. Quantification of virus released into the supernatant was performed by titration in BHK cells. Data represent Mean ± SD of triplicate infection experiments. Dashed lines: detection limit.

The 3′ UTRs of CHIKV belonging to the IO lineage contain two DR elements, namely DR1 (two copies) and DR2 (three copies) [22,26]. To analyze the importance of DR elements in viral replication and vector competence, we rescued CHIKV encompassing deletion of the DR elements 1a + 2a using reverse genetics (CHIKV-∆DR) (Figure 1A). As backbone the CHIKV E1-A226V variant was chosen. This variant is known to better infect *Ae. albopictus* which has also invaded European regions [12,44], and we aimed to analyze especially the vector competence of mosquito species prevalent in temperate regions.

Plaques formed by CHIKV-∆DR were similar to those of CHIKV-WT (Figure 1B). To further analyze the growth of CHIKV-∆DR in cell culture, we infected different cells with mutant and wild-type CHIKV for growth kinetic analyses. Both viruses readily replicated in vertebrate BHK cells with comparable growth characteristics reaching a titer at 44 h post infection of around 5 × 10^8^ (Figure 1C). Efficient growth was also observed in *Ae. albopictus*-derived C6/36 cells with CHIKV-WT growing nearly one log higher than CHIKV-∆DR (Figure 1D). Although CHIKV grew less efficiently in *Ae. aegypti*-derived Aag2 cells, viral titers were also reduced for CHIKV-∆DR compared to CHIKV-WT at later time points post infection (Figure 1E). These growth characteristics could also be demonstrated using reporter viruses of both variants expressing mCherry within the nsP3 protein (Figure S2) [37].

### 3.2. Vector Competence of CHIKV-∆DR in Ae. aegypti and Ae. albopictus

**Figure 2:**
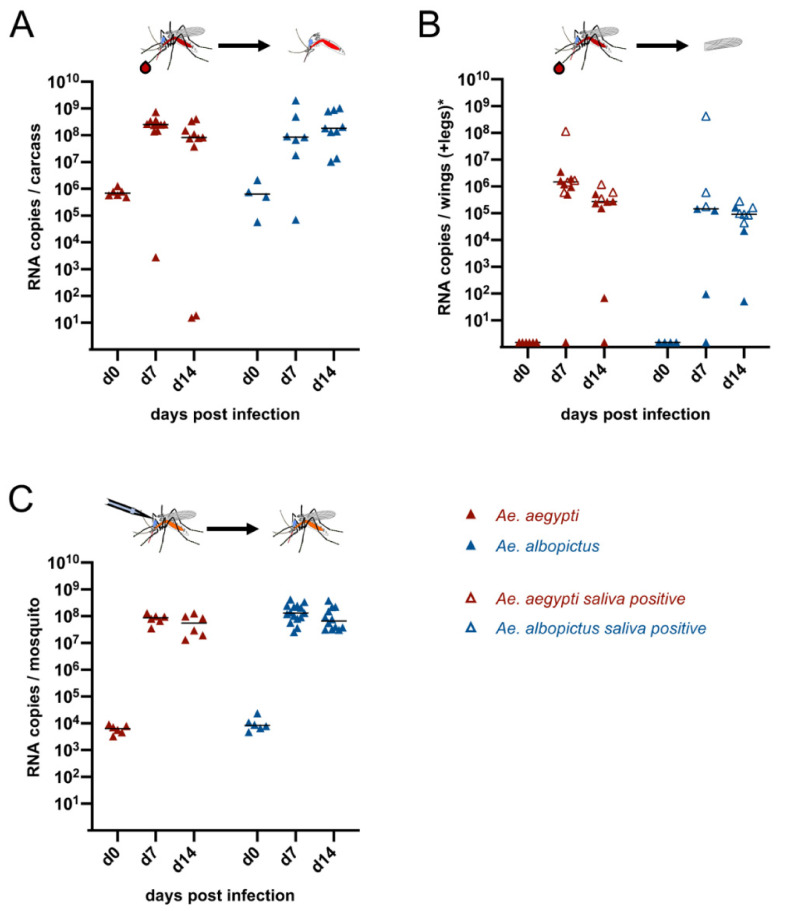
Infection of *Ae. aegypti* and *Ae. albopictus* with CHIKV-∆DR. (**A**) Infection of mosquitoes after oral feeding with blood meal containing 1 × 10^6^ PFU/mL CHIKV-∆DR. At 0, 7 and 14 days post infection, mosquitoes were dissected and viral RNA copies were determined in the carcasses of the mosquitoes by real-time RT-PCR. (**B**) Dissemination of virus into secondary tissues after oral infection. At the indicated time points, viral RNA copies in wings and legs (*Ae. aegypti*) or wings (*Ae. albopictus*) were determined by real-time RT-PCR. Open symbols indicate disseminated mosquitoes for which transmission into saliva was also detected. (**C**) Intrathoracic infection of mosquitoes using 200 PFU of CHIKV-∆DR. At the indicated time points viral load in the whole mosquitoes was determined using real-time RT-PCR. Each point represents a single female mosquito. The black line indicates the median of the viral RNA copies in each group.

*Ae. aegypti* and *Ae. albopictus* represent known vectors for CHIKV. To confirm that the CHIKV-∆DR mutant can also replicate in these mosquitoes, we first infected female *Ae. aegypti* and *Ae. albopictus* orally with the mutant virus using blood meal containing 1 × 10^6^ PFU/mL virus (Figure 2A,B). Mosquitoes were dissected either within three hours after feeding (d0) or at d7 or d14 post infection, respectively. Analyses of mosquito carcasses revealed that viral RNA could be detected in all analyzed mosquitoes resulting in infection rates of 100% (Figure 2A, Table 2). The median viral titers increased between d0 and d7 for both *Ae. aegypti* and *Ae. albopictus* indicating that both mosquito species can efficiently be infected orally with the CHIKV-∆DR virus (Figure 2A). High median viral titers were sustained up to d14.

To analyze for dissemination, the wings and legs of *Ae. aegypti* were pooled and viral RNA copies were measured. For *Ae. aegypti*, the dissemination rate was at least 90% at d7 or d14, respectively (Table 2). Since *Ae. albopictus* were orally fed using cotton swabs instead of a Hemotek, the legs might have been potentially contaminated with virus from the blood meal. Hence, for *Ae. albopictus* only wings were taken to measure the ability of dissemination, which was determined to be 85.7% at d7 or 100% at d14, respectively (Figure 2B, Table 2). To determine whether disseminated virus was also able to reach the salivary glands, we collected the saliva of the mosquitoes and analyzed the samples again via RT-PCR. Transmission could be observed at d7 for 4 out of 10 disseminated *Ae. aegypti* (transmission rate 40%) or at d14 for 3 out of 9 disseminated *Ae. aegypti* (transmission rate 33.3%), respectively (Figure 2B, Table 2). Overall, this yielded transmission efficiencies of 36.4% (d7) or 30% (d14). For *Ae. albopictus* the transmission rates of 50% (d7) and 66.6% (d14), respectively, were obtained resulting in transmission efficiencies of 42.9% (d7) and 66.7% (d14) (Table 2).

While the median infectivity titers increased after oral infection for both *Ae. aegypti* and *Ae. albopictus*, individual *Ae. aegypti* showed only a low viral titer at d7 or d14 post infection in the carcasses (Figure 2A). We therefore also analyzed for *Ae. aegypti* as well as for *Ae. albopictus* as to which extent virus replication occurs when circumventing the midgut barrier. To this end *Ae. aegypti* and *Ae. albopictus* were intrathoracally infected with 200 PFU of CHIKV-∆DR. Viral RNA levels were analyzed at d0 from whole mosquitoes to verify the viral input level and at d7 and d14 post infection to analyze for viral replication. As can be seen in Figure 2C, the median titers increased for all d7 and d14 samples compared to the d0 time point demonstrating that CHIKV-∆DR replicates efficiently in *Ae. aegypti* and *Ae. albopictus* after circumventing the midgut barrier. This suggests that individual *Ae. aegypti* with low viral titers in the carcasses had insufficient oral virus uptake.

### 3.3. Vector Competence of CHIKV-∆DR and CHIKV-WT in Ae. vexans and Cx. pipiens

**Figure 3:**
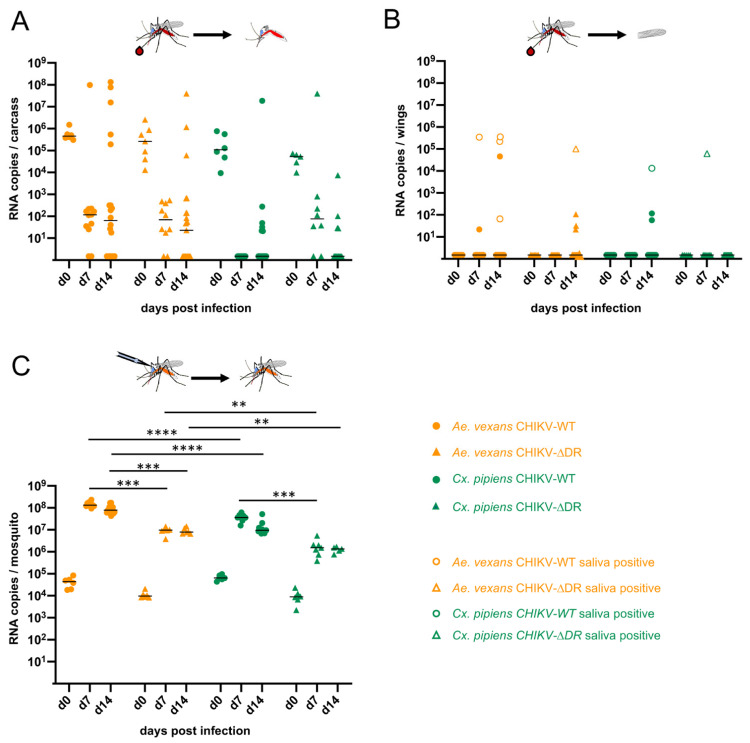
Infection of *Ae. vexans* and *Cx. pipiens* with CHIKV-WT and CHIKV-∆DR. (**A**) Infection of mosquitoes after oral feeding with blood meal containing 1 × 10^6^ PFU/mL CHIKV-WT or CHIKV-∆DR. At d0, d7, and d14 post infection, mosquitoes were dissected and viral RNA copies were determined in the carcasses of the mosquitoes by real-time RT-PCR. (**B**) Dissemination of virus into secondary tissues after oral infection. At the indicated time points, viral RNA copies in the wings were determined. Open symbols indicate disseminated mosquitoes for which also transmission into saliva was detected. (**C**) Intrathoracic infection of mosquitoes using 200 PFU of CHIKV-WT or CHIKV-∆DR. At the indicated time points viral load in the whole mosquitoes was determined. Each symbol represents a single female mosquito. The black line indicates the median of the viral RNA copies in each group (** *p* < 0.01; *** *p* < 0.001; **** *p* < 0.0001).

*Ae. albopictus* is known to have invaded areas of Europe [44]. To analyze whether other mosquito species in temperate regions are also competent for CHIKV and whether DR elements in the 3′ UTR have an impact on possible infection, dissemination and transmission rates, we performed infection experiments with *Ae. vexans* and *Cx. pipiens* (Figure 3).

Female *Ae. vexans* and *Cx. pipiens* were orally feed with a blood meal containing 1 × 10^6^ PFU/mL CHIKV-WT or CHIKV-∆DR, respectively (Figure 3A,B). Analysis of the viral titers in the *Ae. vexans* carcasses at d7 and d14 post infection revealed infection rates for CHIKV-WT of 83.3% (d7) and 65% (d14), and for CHIKV-∆DR of 80% (d7) and 47.8% (d14), respectively (Figure 3A, Table 3). To assess whether infected *Ae. vexans* are able to disseminate the viruses, wings were analyzed for genomic RNA of CHIKV (Figure 3B). At the early time point of 7 days post infection, the dissemination rate for CHIKV-WT was 20% at d7 and increased to 30.8% at d14. An increase of the dissemination rate over time was also observed for CHIKV-∆DR (0%, d7 and 36.4%, d14) (Table 3). Transmission rates in *Ae. vexans* were in the range of 50–75% at d7 or d14 for CHIKV-WT, whereas only a transmission rate up to 25% was observed for CHIKV-∆DR at d14 post infection. Altogether, this resulted in transmission efficiencies of 8.3% (d7) and 15% (d14) for CHIKV-WT in *Ae. vexans*, which however were not significantly higher than the transmission efficiencies of CHIKV-∆DR with 0% (d7) and 4.3% (d14).

In case of *Cx. pipiens*, viral RNA could be detected after oral feeding in the mosquito carcasses to 0% (d7) and 30% (d14) for CHIKV-WT and 75% (d7) and 28.5% (d14) for CHIKV-∆DR (Figure 3A and Table 3). Dissemination occurred rather sporadically and was found in three mosquitoes infected with CHIKV-WT at d14 and one mosquito infected with CHIKV-∆DR at d7 post infection (Figure 3B, Table 3). Viral RNA in the saliva could be detected for the two mosquitoes where the viral load in the wings was above 1 × 10^4^ RNA copies, namely once for CHIK-WT (d14) and once for CHIKV-∆DR (d7) suggesting a low potential for transmission (Figure 3B, Table 3).

For both *Ae. vexans* as well as *Cx. pipiens* it was observed that once increased viral titers were found in the carcasses, the virus was usually also able to disseminate and reach the salivary glands. This suggested that the midgut barrier represents the main barrier with regard to vector competence. To further support this finding, we infected *Ae. vexans* and *Cx. pipiens* intrathoracally using again 200 PFU with CHIKV-WT or CHIKV-∆DR, respectively. As can be seen in Figure 3C, analyses of the whole mosquitoes revealed efficient virus replication. CHIKV-WT replicated significantly higher in *Ae. vexans* compared to CHIKV-∆DR at both d7 and d14 post infection (*p* = 0.0002 or *p* = 0.0003, respectively). For *Cx. pipiens* a significant difference for CHIKV-WT in comparison to CHIKV-∆DR was only observed at d7 post infection (*p* = 0.0037). Furthermore, differences were obtained comparing mosquito species. CHIKV-WT replicated significantly higher in *Ae. vexans* compared to *Cx. pipiens* (*p* < 0.0001 for d7 and d14) and also replication of CHIKV-∆DR resulted in significant differences at d7 (*p* = 0.0023) and d14 (*p* = 0.0043). Furthermore, significant differences were obtained comparing the viral loads of CHIKV-∆DR after intrathoracic injection in *Ae. aegypti* (Figure 2) to the ones obtained in *Cx. pipiens* (*p* = 0.0012, d7 and *p* = 0.0043, d14). The differences were even more significant comparing the replication rates of CHIKV-∆DR after intrathoracic injection of *Ae. albopictus* (Figure 2) with the ones of *Cx. pipiens* (*p* < 0.0001, d7 and *p* < 0.0003, d14) suggesting that replication rates in the secondary tissues differ among both mosquito species and genus.

## 4. Discussion

The presence of repetitive elements in alphaviruses was already described over 20 years ago [20,21,45]. The number and arrangement of these direct repeat elements varies not only among different alphavirus species but also within species. In the Venezuelan equine encephalitis virus complex as well as for Ross River virus, isolates from mosquitoes in nature with various numbers of repeat elements have been described but their impact on ecology and pathogenesis is not well understood [21,46]. Comparison of the CHIKV genotypes likewise revealed various arrangements of repeat elements within the 3′ UTR [22]. The authors suggested that these differences were a result of an evolutionary process in order to especially adapt to mosquitoes rather than to host species. We investigated whether deletion of the DR elements DR1a and DR2a in CHIKV Mauritius belonging to the IO lineage affects viral replication in cell cultures or mosquitoes. While growth in vertebrate BHK cells was not affected for the mutant, the deletion resulted in reduced growth in *Ae. albopictus* mosquito cells. Similar results were already described many years ago for a Sindbis virus (SINV) deletion mutant and more recently for CHIKV deletion mutants of the Asian genotype as well as the IO lineage [22,26,47,48]. For SINV, viable virus was still recovered after removing up to 293 nt in the 3′ UTR so that only 25 nt from the 3′ terminus of the genome including the CSE element and one nucleotide 3′ of the UGA termination codon of the structural proteins was retained [47]. The resulting SINV mutant grew less efficiently in mosquito cells than in vertebrate cells and was in general more impaired than variants with smaller 3′ UTR deletions. Deletion of different numbers and sets of DR elements based on a Caribbean CHIKV isolate also resulted in reduced growth in mosquito C6/36 cells, whereby the growth was more restricted for the mutant with the larger deletion [26]. The same authors also deleted both the DR1ab and DR2ab motifs in CHIKV La Reunion belonging also to the IO lineage. This decreased the growth in C6/36 cells of about 3–4 logs while deletion of only DR1a and DR2a in our Mauritius isolate resulted in a reduction of about only 1 log supporting the finding that the size of deletion correlates with the level of growth reduction.

Although *Ae. aegypti* is known to be a main vector for CHIKV, *Ae. aegypti* derived Aag2 cells did not support the replication of CHIKV Mauritius as efficiently as C6/36 cells. After infection with the same MOI of 0.1, increase of viral titers was delayed and only peaked around 10^5^ PFU/mL. Other studies also describe a comparatively low replication of CHIKV in Aag2 cells [49]. The efficiency of CHIKV release from Aag2 cells in comparison to C6/36 cells also seems to vary between different CHIKV isolates. Viral RNA copies in the supernatant of Aag2 cells increased faster within 24 h for a CHIKV belonging to the Asian genotype compared to CHIKV of the IO lineage belonging to the ECSA genotype [50]. Nevertheless, as observed for C6/36 cells, replication of CHIKV-∆DR was also impaired in Aag2 cells. As discussed previously, the phenotypic differences observed between vertebrate and mosquito cells might be attributed to different 3′ UTR binding proteins [47]. In line with this hypothesis, functional differences between 3′ UTRs in vertebrate and insect cells were described with regard to translation. A motif of repetitive sequence elements in the 3′ UTR was found to be involved in translation of SINV genomic and subgenomic mRNAs in mosquito cells but not in vertebrate cells [51]. It was discussed that such repetitive motifs might have been acquired during evolution to extend the host range towards mosquitoes [51].

Despite the fact that the deletion mutant showed reduced growth in mosquito cells compared to its wild-type counterpart, it readily could infect and disseminate in the well-known mosquito vectors *Ae. aegypti* and *Ae. albopopictus* both after oral and intrathoracic infection. Recent studies based on a Caribbean isolate compared a 3′ UTR mutant with several DR elements deleted to the wild-type virus with regard to infection, dissemination and transmission rates in *Ae. aegypti* and *Ae. albopictus* mosquitoes [27]. These analyses showed that the wild-type virus had a shorter extrinsic incubation period and it was found that the replication kinetics determine the ability to escape from the midgut. The slower replicating viruses encompassing the 3′ UTR deletion were less able to cross the midgut barrier as was shown in competition experiments with the wild-type virus [27]. Increased viral titers were also observed for the wild-type virus compared to the deletion mutant after intrathoracic infection but only at the earlier time points after infection (d2 and d4) [27]. This is in accordance with our findings that no significant differences between mutant and wild-type virus were obtained after intrathoracic injection in *Ae. vexans* or *Cx. pipiens*, respectively, when analyzing samples from d7 and d14 post infection.

However, statistically significant differences were obtained when comparing one type of virus in different mosquito species. After intrathoracic infection with CHIKV-∆DR the viral titers increased by 4 logs in both *Ae. aegypti* and *Ae. albopictus* (Figure 2C) whereas an increase of only 3 or 2 logs was observed for *Ae. vexans* or *Cx. pipiens*, respectively (Figure 3C). Similarly, the intrathoracically injected CHIKV-WT replicated to higher titers in *Ae. vexans* compared to *Cx. pipiens*. Hence, the midgut escape barrier does not seem to be the only factor influencing vector competence of mosquitoes. Also, replication kinetics in the secondary tissues seem to differ between mosquito species.

Both factors would also result in a longer extrinsic incubation period of CHIKV in *Ae. vexans* and *Cx. pipiens* compared to *Ae. aegypti* and *Ae. albopictus* after oral infection. This is supported by the fact that for *Ae. vexans* high viral titers within the body were not observed until d14, the time point where also the majority of mosquitoes with disseminated virus was observed (Figure 3A,B). Furthermore, detection of virus in the saliva was mainly observed in samples with high viral load in the wings suggesting that efficient replication in the secondary tissues to reach a certain threshold is critical for transmission. Although less *Cx. pipiens* mosquitoes were infected after oral feeding compared to *Ae. vexans*, dissemination as well as transmission was observed when 10^4^ or more RNA copies were detected in the wings. This indicates that *Cx. pipiens* potentially could serve as a vector. To our knowledge this is the first study analyzing the vector competence of *Cx. pipiens* from Germany for CHIKV. Previous studies investigated the vector competence of *Ae. vexans* and *Cx. pipiens* using mosquitoes hatched from larvae or pupae sampled in France or Italy [52,53]. Whereas *Ae. vexans* from France as well as *Cx. pipiens* from both France and Italy were refractory to CHIKV infection, a low disseminated infection rate was observed in *Ae. vexans* from Italy based on immunofluorescence analyses of head squashes [52,53]. Analyses of another *Aedes* species, namely *Aedes geniculatus* originating from Albania revealed that they are susceptible to CHIKV and could transmit the virus although a longer extrinsic incubation period was observed compared to *Ae. albopictus* [54]. CHIKV positive *Cx. pipiens* were also detected during an outbreak in the Amazon region of Bazil. Since only mosquito pools were analyzed using PCR, no statement on the transmission rate could be made [55]. Nevertheless, our results indicate that other mosquito species than the well-established *Ae. aegypti* and *Ae. albopictus* may serve as competent mosquito vectors not only under laboratory conditions.

## 5. Conclusions

Our results demonstrate that the well-known CHIKV vectors *Ae. aegypti* and *Ae. albopictus* are also competent vectors for CHIKV exhibiting a two DR element deletion in the 3′ UTR. Testing two mosquito species, *Ae. vexans* and *Cx. pipiens*, for which little data on vector competence is currently available, demonstrated single mosquito individuals for which both CHIKV-WT and CHIKV-∆DR were able to overcome the midgut infection barrier and appeared in the saliva 7 or 14 days after oral infection. Together with the finding that these mosquito species replicated CHIKV-WT and CHIKV-∆DR in secondary tissue very efficiently after intrathoracic infection, both mosquito species may eventually transmit CHIKV also in nature.

## Figures and Tables

**Table 1:** Taxa and origin of mosquitoes used in the infection experiments.

Mosquito Taxon	Origin/Collection Site	Year of Collection	Year (Generation) Colony Established in Leipzig	Generations Used for Experiments
*Aedes aegypti*	Geigy, Switzerland ^1^	1950	2017 (F1) ^2^	F2–F5
*Aedes albopictus*	Cesena, Italy	2016	2016 (F2)	F11–F13
*Aedes vexans ‘TAMU’*	Texas, USA	2002	2013 (F35)	F45–F53
*Culex pipiens* biotype *molestus*	Hesse, Germany	2000	2013 (F1) ^2^	F55–F85

^1^ crossed with strain from BASF, Ludwigshafen, Germany in 1972 and again with strain from Biogents AG, Germany in 2017. ^2^ No data on the filial generation was available. Therefore, numbering for the colony in Leipzig was started with F1.

**Table 2:** Infection, dissemination, transmission rates, and transmission efficiencies for CHIKV-∆DR at d7 and d14 post infection in *Ae. aegypti* and *Ae. albopictus.*

Mosquito Species	Virus	Days Post Infection (%)	Infection Rate (%)	Dissemination Rate (%)	Transmission Rate (%)	Transmission Efficiency (%)
*Aedes aegypti*	∆DR	7	11/11	10/11	4/10	4/11
			100%	90.9%	40%	36.4%
		14	10/10	9/10	3/9	3/10
			100%	90%	33.3%	30%
*Aedes albopictus*	∆DR	7	7/7	6/7	3/6	3/7
			100%	85.7%	50%	42.9%
		14	9/9	9/9	6/9	6/9
			100%	100%	66.6%	66.7%

**Table 3:** Infection, dissemination, transmission rates, and transmission efficiencies for CHIKV-WT and CHIKV-∆DR at d7 and d14 post infection in *Ae. vexans* and *Cx. pipiens.*

Mosquito Species	Virus	Days Post Infection (%)	Infection Rate (%)	Dissemination Rate (%)	Transmission Rate (%)	Transmission Efficiency (%)
*Aedes vexans*	WT	7	10/12	2/10	1/2	1/12
			83.3%	20%	50%	8.3%
		14	13/20	4/13	3/4	3/20
			65%	30.8%	75%	15%
	∆DR	7	8/10	0/8	0/0	0/10
			80%	0%	n.a.	0%
		14	11/23	4/11	1/4	1/23
			47.8%	36.4%	25%	4.3%
*Culex pipiens*	WT	7	0/9	0/0	0/0	0/9
			0%	n.a.	n.a.	0%
		14	6/20	3/6	1/3	1/20
			30%	50%	33.9%	5%
	∆DR	7	6/8	1/6	1/1	1/8
			75%	16.6%	100%	12.5%
		14	4/14	0/4	0/0	0/14
			28.5%	0%	n.a.	0%

## Supplementary Materials

The following are available online at https://www.mdpi.com/1999-4915/13/3/403/s1, Figure S1: CHIKV real-time PCR; Figure S2: Expression of mCherry from CHIKV-WT and CHIKV-ΔDR reporter viruses at different time points after infection.
