# Supplementary material for: Characterization and Vector Competence Studies of Chikungunya Virus Lacking Repetitive Motifs in the 3′ Untranslated Region of the Genome
Source: Viruses. 2021 Mar 4;13(3):403. doi: 10.3390/v13030403 (PMC7999577; doi:10.3390/v13030403)
Supplement: Supplementary file 1 [file viruses-13-00403-s001.pdf]

Supplementary Material

A

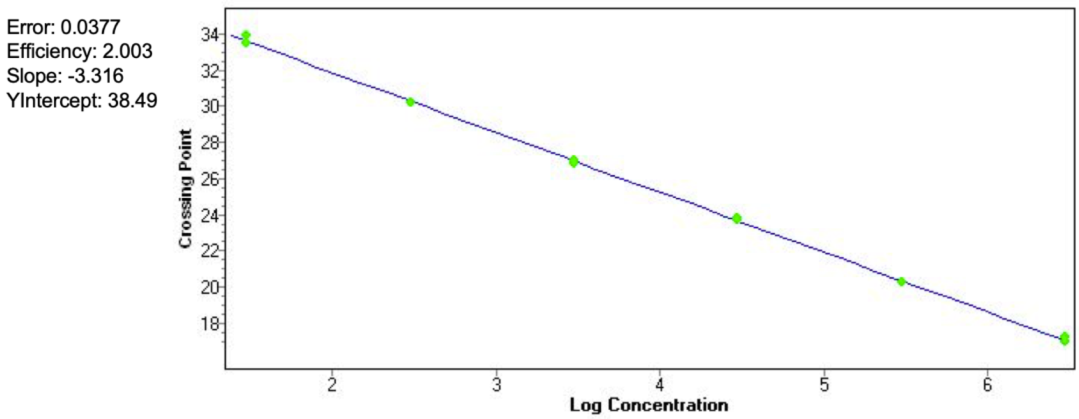

B

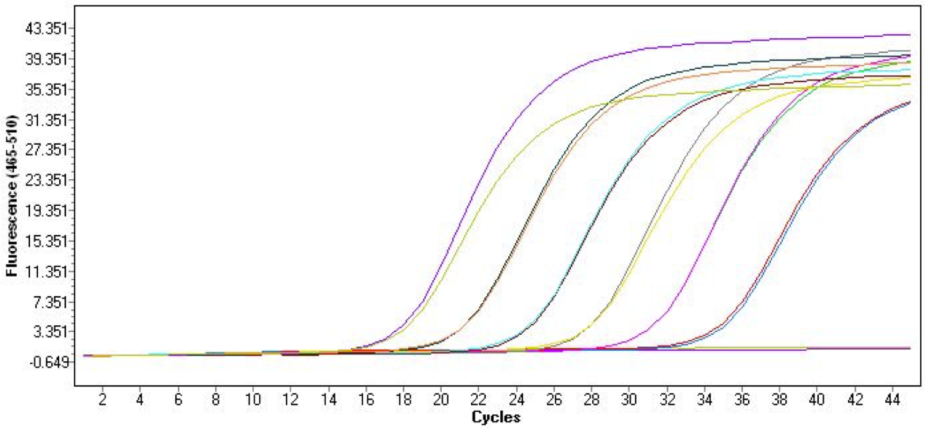

Figure S1: CHIKV real-time PCR. A: Standard curve of threshold cycle number plotted against the log of initial template concentrations in RNA copies. Shown is one representative standard curve. Data represent duplicates of each dilution. The ten-fold serial dilutions are linear over six logs. B: Amplification plot of the same experiment showing the ten-fold dilution series of RNA transcripts. No amplification was observed in a control reaction.

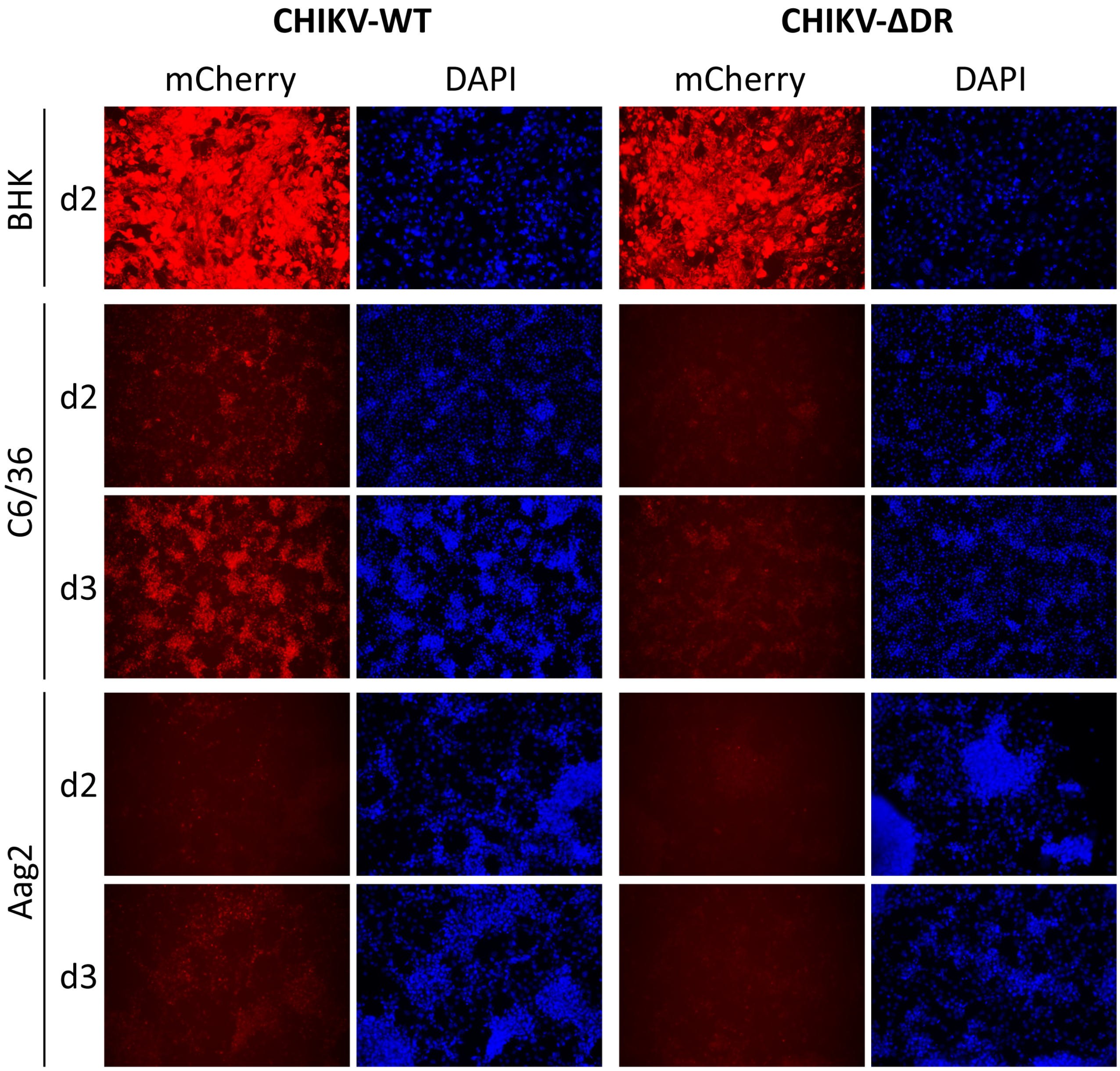

Figure S2. Expression of mCherry from CHIKV-WT and CHIKV-ΔDR reporter viruses at different time points after infection. BHK, C6/36, and Aag2 cells were infected with CHIKV-WT-mCherry or CHIKV-ΔDR-mCherry at an MOI of 0.1. At the indicated time points cells were fixed and stained with DAPI. Pictures demonstrate individual fluorescence of mCherry or DAPI, respectively.

Supplementary Experimental Procedure

Cloning and rescue of CHIKV-mCherry reporter viruses

To obtain the CHIKV reporter viruses expressing mCherry inserted into nsP3, pCHIKV-mCherry-490 [1] was cut with *Age*I and *Ngo*MIV. The fragment encompassing the mCherry gene was inserted into pCHIKV-WT or pCHIKV-ΔDR cut with the same restriction enzymes. Recombinant viruses were rescued as described in the main manuscript.

Reference

1. Kümmerer, B. M.; Grywna, K.; Gläsker, S.; Wieseler, J.; Drosten, C., Construction of an infectious Chikungunya virus cDNA clone and stable insertion of mCherry reporter genes at two different sites. *J Gen Virol* **2012,** 93, (Pt 9), 1991-1995.
